# Self-esteem and psychological well-being as predictors of life satisfaction among university students in southern Peru: a cross-sectional predictive study

**DOI:** 10.1186/s40359-026-05212-y

**Published:** 2026-07-20

**Authors:** Paola Mercedes Mayhua Taco, María Inés Apaza Chambi, Yesenia Estefany Pelinco Diaz, Lucy Puño-Quispe

**Affiliations:** Escuela Profesional de Psicología, Facultad de Ciencias de la Salud, Universidad Peruana Unión, Juliaca, Perú

**Keywords:** Self-esteem, Psychological well-being, Life satisfaction, Psychological health, University students, Psychological distress, Social bonds

## Abstract

**Background:**

Self-esteem and psychological well-being are fundamental pillars of individuals’ integral development, especially during the university stage, when students face multiple personal challenges and intense academic demands.

**Objective:**

This study aimed to determine whether self-esteem and psychological well-being predicted life satisfaction among university students in southern Peru.

**Method:**

The sample of 506 university students, aged 18 to 30 years *(M = 22.1; SD = 2.84)*, participated in this cross-sectional predictive study. The data were collected from three universities in southern Peru via non-probabilistic convenience sampling. The instruments were the Rosenberg Self-Esteem Scale, the BIEPS-A Psychological Well-Being Scale for Adults, and the Satisfaction with Life Scale. Hierarchical regression analysis was performed.

**Results:**

Hierarchical regression showed that psychological well-being dimensions (*ΔR²=0.426*) and self-esteem (*ΔR²=0.157*) significantly predicted life satisfaction, explaining 59.6% of the variance, while sociodemographic variables showed no significant contribution (*R²=0.013*,*p* = 0.240). Self-esteem was the strongest predictor in the final model (unstandardized *B = 0.363*,* SE = 0.026*,* β = 0.572*,*p* < 0.001), followed by the psychological well-being dimension of social bonds (*B = 0.424*,*SE = 0.087*,*β = 0.185*,*p* < 0.001).

**Conclusion:**

The self-esteem and psychological well-being dimensions are significant predictors of life satisfaction among university students in southern Peru, with self-esteem also operating as a mediating mechanism linking psychological well-being to life satisfaction.

## Introduction

University life constitutes a critical period in young adults’ psychological development, as intense academic, social, and personal demands can significantly affect their mental health and overall well-being [1]. Mental health problems affect up to 50% of higher education students worldwide, and student well-being indices remained critically low even after the COVID-19 pandemic, confirming that university students’ mental health remains in crisis beyond this period [2], with approximately 20% reporting depressive symptoms, 15% experiencing suicidal ideation, and between 2% and 3% having made suicide attempts [3]. In the United States, university students’ mental health has become a major concern for educational authorities, as reports from 2023 to 2024 documented a noticeable increase in psychological and emotional difficulties among this population [4]. In Latin America, a study revealed that approximately one in seven individuals experiences some form of anxiety disorder at some point in life [5]. The Global Happiness Report by Ipsos also noted declines in life satisfaction and mental health among Generation Z, raising concerns about their psychological well-being [6].

In Peru, 32.3% of young people aged 15 to 29 reported experiencing some form of mental health problem. Additionally, 23% stated that they had felt discouraged, sad, or hopeless, and 5.6% indicated having had thoughts of death or self-harm [7]. These challenges are particularly pronounced in structurally disadvantaged contexts such as southern Peru, characterized by geographic barriers, economic constraints, high rates of alcohol consumption among young people, limited access to mental health services, and marked disparities in health access, all of which may influence the psychological adjustment and subjective well-being of university students in the region [8–10]. These structural conditions are not merely descriptive; they offer a theoretical basis for why the relationship among self-esteem, psychological well-being, and life satisfaction might operate distinctively in this context. Self-determination theory [11] holds that psychological well-being depends on satisfying basic needs for autonomy, competence, and relatedness, which are typically supported by stable external conditions such as economic security and accessible mental health services. Where such conditions are scarce, as in southern Peru, individuals may come to rely more heavily on internal resources, such as self-esteem, to sustain a positive appraisal of their lives, consistent with evidence that self-esteem mediates the effects of perceived poverty on life satisfaction in other under-resourced student populations [12]. This offers a theoretically motivated, region-specific rationale for why self-esteem might carry comparatively greater weight in shaping life satisfaction in this context, moving beyond treating southern Peru merely as a geographic setting in which previously established relationships are re-examined, and toward a context whose structural conditions may themselves help account for the expected pattern of results.

Within this context, two psychological constructs have emerged as central determinants of life satisfaction: self-esteem and psychological well-being. Diener’s model conceptualizes life satisfaction as the cognitive evaluative component of subjective well-being [13, 14]. It is important to distinguish psychological well-being from clinical indicators of psychological disorders: Ryff’s model [15] refers to positive psychological functioning, purpose, autonomy, self-acceptance, personal growth, environmental mastery, and positive relations, which are conceptually distinct from psychopathology. Ryff’s model complements Diener’s framework by demonstrating that psychological functioning contributes directly to positive life evaluations [16].

Self-esteem is a key determinant of the personal development of university students, as it contributes to higher levels of life satisfaction, promotes emotional well-being, and facilitates adjustment to academic demands [17]. It functions as a stable psychological resource influencing emotional regulation, coping strategies, and cognitive appraisals of life circumstances [12]. Sustaining high self-esteem not only enhances well-being and encourages initiative but also supports success and the cultivation of personal virtues [18]. Longitudinal evidence suggests that individuals with higher self-esteem maintain positive well-being across multiple life domains [19], and higher levels of self-esteem have been consistently associated with enhanced quality of life and greater personal fulfillment [20, 21]. Evidence from Israel further revealed that university students who manage their emotions effectively and find a sense of meaning in life tend to feel calmer, happier, and more satisfied [22].

Various studies have reported a positive association among self-esteem, psychological well-being, and life satisfaction in university students. Enhancing self-esteem and resilience may also serve as protective factors, buffering the negative impact of early adverse experiences and contributing to greater happiness in young people [23]. A study conducted with 243 university students enrolled in a psychology program in Malaysia found a strong positive relationship between self-esteem and life satisfaction [24]. In Ecuador, a study of 476 university students found that subjective well-being, comprising positive affect, negative affect, and life satisfaction, was significantly associated with mental health, with results demonstrating gender invariance and suggesting that subjective well-being plays a relevant role in the development of mental health in this population [25]. A study conducted with Peruvian university students showed that psychological well-being facilitates the development of healthy social relationships, supports growth, and contributes meaningfully to better academic performance [26]. Although these studies converge in highlighting the relevance of self-esteem and psychological well-being for life satisfaction, most rely on cross-sectional designs that preclude causal inference, and few have examined these relationships within socioculturally disadvantaged contexts such as southern Peru. In this regard, identifying these psychological predictors is essential for designing effective interventions to promote mental health and academic success among university populations [27–30].

It is important to acknowledge that the theoretical directionality of these relationships is not uniformly established in the literature. While most studies position self-esteem and psychological well-being as antecedents of life satisfaction, some studies have proposed inverse or reciprocal pathways, suggesting that global evaluations of life may, in turn, influence psychological functioning. This bidirectionality is theoretically plausible and has been recognized in the broader literature on subjective well-being [14]. The present study is theoretically grounded in Diener’s subjective well-being framework [13, 14] and Ryff’s psychological well-being model [15], which together support the proposition that self-esteem and psychological well-being function as stable internal resources that precede and shape an individual’s cognitive evaluations of their overall life satisfaction [16, 19]. Nevertheless, given the cross-sectional design of this study, causal directionality cannot be definitively established; bidirectional or inverse relationships should be considered when interpreting the findings.

Therefore, the objective of this research was to determine whether self-esteem and psychological well-being predict life satisfaction among university students in southern Peru. Based on the theoretical frameworks of Diener and Ryff, two hypotheses were formulated: H_1_: self-esteem and psychological well-being will significantly and positively predict life satisfaction among university students in southern Peru. H_2_: Self-esteem will mediate the relationship between the psychological well-being dimensions and life satisfaction, such that the indirect effects of well-being dimensions on life satisfaction through self-esteem will be statistically significant.

## Method

The study is empirical, with a predictive and cross-sectional design [31]. The term predictive refers to the use of regression-based statistical methods to examine the relative contribution of self-esteem and psychological well-being dimensions to variance in life satisfaction, and does not imply causal directionality given the cross-sectional nature of the design.

### Participants

The sample size was determined through an a priori calculation using the A-priori Sample Size Calculator for Multiple regression [32]. A medium effect size was established (*f*^*2*^ = 0.15), with *α = 0.05*, a desired statistical power of 0.95, and two predictors. The analysis indicated that the minimum required sample size was 106 participants, the number needed to detect the anticipated effect. A total of 506 participants were ultimately recruited, exceeding the required minimum and ensuring a 95% probability of detecting a medium effect if it truly exists. Participants were selected via non-probabilistic convenience sampling, in which individuals were included based on their accessibility and willingness to participate. Although convenience sampling limits external validity, it was deemed appropriate given the study’s exploratory nature and the limited accessibility of university students in southern Peru, a region characterized by geographic, economic, and structural barriers that constrain probabilistic sampling. Nonetheless, because participation required access to the internet and a smartphone, students from lower-income backgrounds or without reliable connectivity in rural areas may have been underrepresented. Consequently, the findings should be interpreted as contextually situated evidence applicable to university students in southern Peru, and caution is warranted when generalizing to other regional or national university populations. The sample comprised 506 university students aged 18 to 30 years *(M = 22.1; SD = 2.84)*, of whom 269 (53.2%) were women. Regarding institutional affiliation, the majority were enrolled at public universities (*n* = 353, 69.8%), while 153 (30.2%) attended private institutions. Regarding place of residence, most participants reported living in urban areas (*n* = 403, 79.6%) and rural areas (*n* = 103, 20.4%). Although sociodemographic characteristics, including age, gender, marital status, field of study, semester, and place of residence, were collected and are presented in Table 1, socioeconomic status was not assessed, which constitutes a limitation given its relevance as a contextual variable in Peruvian university studies.

### Instruments

To assess the first variable, the Rosenberg Self-Esteem Scale, originally developed by Rosenberg and adapted to the Peruvian context by Robles [33], was used. The instrument is unidimensional, and in terms of construct validity, the scale demonstrated adequate fit indices (RMSEA = 0.059, CFI = 0.950, and TLI = 0.933). The reliability coefficient was 0.797, indicating good internal consistency. The scale consists of 10 items, of which five are positively worded [1, 4, 5, 24, 26] and five are negatively worded [3, 6, 7, 23, 25]. Responses are rated on a Likert-type scale (1 = strongly agree, 2 = agree, 3 = disagree, 4 = strongly disagree), and total scores are categorized into four levels: 0–10 low, 11–20 medium-low, 21–30 medium-high, and 31–40 high [33].

To assess the second variable, the Psychological Well-being Scale (BIEPS-A) was used, which was adapted to the Peruvian context by Domínguez [34, 35]. The instrument consists of 13 items distributed across four dimensions: acceptance/control of situations (items 2, 11, and 13), autonomy (items 4, 9, and 12), projects (items 1, 3, 6, and 10), and social bonds (items 5, 7, and 8). Responses are provided on a Likert-type scale (1 = disagree), (2 = neither agree nor disagree), and (3 = agree). The scale demonstrated satisfactory fit indices (CFI = 0.9613, GFI = 0.8957, RMR = 0.032, RMSEA = 0.0919), with reliability coefficients of *ω = 0.706* for acceptance/control, *ω = 0.765* for autonomy, *ω = 0.864* for projects, and *ω = 0.833* for social bonds. Levels are classified as low [17–35], medium [36–44], and high [8, 9, 32, 45–50].

To assess the third variable, the Satisfaction with Life Scale (SWLS) was used, which was validated in the Peruvian context by Arias-Chávez et al. [36]. This unidimensional instrument consists of five items, with responses rated on a Likert-type scale ranging from 1 = Strongly disagree to 5 = Strongly agree. Evidence of validity was obtained through confirmatory factor analysis, which showed a satisfactory fit for the unidimensional structure of the scale (CFI = 0.998; TLI = 0.997; RMSEA = 0.021; SRMR = 0.021). The instrument demonstrated good internal consistency, with a Cronbach’s Alpha of 0.79 and an Omega coefficient of 0.80. Furthermore, the scale includes five levels: very dissatisfied [6–13], dissatisfied [17], neutral [15–20], satisfied [21–33], and very satisfied [34–39]; and percentile classifications defined as low (< 22), medium (22 < 31), and high (≥ 31).

Although the instruments differ in their factorial structure, the Rosenberg Self-esteem and the SWLS are unidimensional, whereas the BIEPS-A is multidimensional; the four BIEPS-A dimensions were entered as separate predictors in all analyses rather than as a global composite score, thereby preserving the instrument’s multidimensional structure and avoiding the loss of differential information that would result from aggregation. This decision was theoretically motivated by Ryff’s proposition that the dimensions of psychological well-being represent distinct functional domains that may contribute differentially to life satisfaction, and that aggregating them into a global score would obscure these differential contributions [15].

### Procedure

Data were collected using a self-administered online questionnaire via Google Forms. The survey link was distributed through social media platforms such as WhatsApp, Instagram, and Facebook, targeting university students enrolled in southern Peru. Of the 556 individuals who accessed the survey link, 506 provided a complete response, yielding a response rate of 90.9%. To safeguard data integrity and prevent duplicate entries, the platforms were configured to allow only one response per authenticated user account. Before completing the questionnaire, all participants received a standardized information sheet outlining the study objectives, the voluntary nature of participation, the right to withdraw at any time without consequence, and the guarantees of anonymity and confidentiality. Informed consent was obtained electronically, requiring each participant to explicitly confirm agreement before proceeding. All data were subsequently exported to Microsoft Excel for initial screening and analyzed using RStudio version 4.5.1.

### Ethical considerations

The study was approved by the Ethics Committee of the Faculty of Health Sciences at Universidad Peruana Unión (approval number 2025-CEB-FCS-UPeU-016). All procedures were conducted in accordance with the ethical principles established in the Declaration of Helsinki. Before data collection, informed consent was obtained from each participant, thereby ensuring voluntary, informed, and confidential participation.

### Data analysis

All analyses were performed in RStudio [51]. Categorical variables (gender, university type, place of residence) were dummy-coded, with “female”, “public university”, and “urban residence” as reference categories. No missing data were present, as the survey platform required all items to be completed before submission. Descriptive statistics, means, standard deviations, and frequency distributions were computed for all study variables and examined separately by gender. Normality of the residuals was assessed using the Shapiro-Wilk test, which indicated a significant deviation from normality (*W = 0.990*,*p* = 0.002). However, considering the relatively large sample size and the well-established robustness of regression analysis to moderate violations of this assumption, this deviation was deemed acceptable. This interpretation was further supported by visual inspection of the histogram and Q-Q plots, which showed no substantial deviations from normality. Analysis proceeded using standard OLS estimation, as the Central Limit Theorem supports approximating sampling distributions as normal in large samples (*n* = 506), and OLS regression is well established as robust to moderate violations of these assumptions under such conditions. No data transformations were applied to the study before analysis. The independence of errors was examined using the Durbin-Watson statistic, which indicated no evidence of autocorrelation (*DW = 2.02*,*p* = 0.892). Multicollinearity diagnostics showed variance inflation factor (VIF) values ranging from 1.63 to 2.04, all well below the commonly accepted threshold of 5, suggesting that multicollinearity was not a concern in the model. Additionally, visual inspection of residual plots indicated that the assumptions of linearity and homoscedasticity were adequately met. To provide a more comprehensive understanding of the relationships among variables, two complementary analytical approaches were employed: hierarchical regression to assess incremental predictive effects and structural equation modeling to test mediation mechanisms.

A hierarchical multiple regression analysis was conducted in three steps. In Model 1, sociodemographic variables were entered as control variables. In Model 2, the four dimensions of psychological well-being (control, autonomy, projects, and social bonds) were included. In Model 3, self-esteem was added to assess its incremental contribution to life satisfaction. For each model, unstandardized (*B*) and standardized (*β*) coefficients, standard errors, confidence intervals, and changes in explained variance *(ΔR²)* were reported.

To examine the mediating role of self-esteem, a path analysis was conducted within a structural equation modeling framework using the *lavaan* package. The model was estimated using maximum likelihood with bias-corrected bootstrap confidence intervals based on 5000 resamples. Model fit was evaluated using CFI, TLI, RMSEA, and SRMR, following conventional cutoff criteria (CFI and TLI ≥ 0.95; RMSEA and SRMR ≤ 0.08). Indirect effects (a × b) were considered statistically significant when the 95% bias-corrected bootstrap confidence intervals did not include zero. Standardized coefficients were reported to facilitate the interpretation of effect sizes. The interpretation of mediation focused on the magnitude and significance of indirect effects rather than on the significance of direct effects alone.

## Results

Table 1 presents the sociodemographic characteristics of the sample. The majority of participants were female (53.2%), single (95.1%), enrolled at public universities (69.8%), and residing in urban areas (79.6%), with engineering students comprising the largest academic group (68.8%).

**Table 1:** Sociodemographic and academic characteristics of Peruvian university students

Variables	M	SD	Characteristics	*n*	%
Age	22.1	± 2.84	18–30	506	100%
Gender			Female	269	53.2%
			Male	237	46.8%
Marital status			Single	481	95.1%
			Married	4	0.8%
			Cohabiting	21	4.2%
			Public university	353	69.8%
			Private university	153	30.2%
Field of study			Faculty of Health Sciences	54	10.7%
			Faculty of Business Sciences	50	9.9%
			Faculty of Engineering and Architecture	348	68.8%
			Faculty of Education	37	7.3%
			Faculty of Social Sciences	17	3.4%
Semester			1	61	12.1%
			2	81	16%
			3	46	9.1%
			4	37	7.3%
			5	47	9.3%
			6	59	11.7%
			7	48	9.5%
			8	127	25.1%
Place of residence			Urban area	403	79.6%
			Rural area	103	20.4%

*n  *absolute frequency,* % *relative frequency,* M *mean,* SD *standard deviation

Table 2 presents the descriptive statistics for the study variables. Notably, nearly half of participants reported low levels of psychological well-being (49.6%), and life satisfaction was predominantly neutral (27.5%), suggesting suboptimal psychological functioning in a substantial proportion of the sample. Self-esteem was comparatively more favorable, with the majority reporting medium-high levels (54.7%).

**Table 2:** Descriptive statistics and levels of life satisfaction, psychological well-Being, and self-esteem among university students

Variables	M	SD	Levels	*n*	%
Life satisfaction	15.92	4.40	Very dissatisfied	105	20.8
			Dissatisfied	29	5.7
			Neutral	139	27.5
			Satisfied	113	22.3
			Very satisfied	120	23.7
Psychological well-being	26.77	5.87	Low	251	49.6
			Medium	108	21.3
			High	147	29.1
Self-Esteem	26.13	6.94	Low	1	0.2
			Medium low	106	20.9
			Medium high	277	54.7
			High	122	24.1

*M *mean,* SD *standard deviation,* n *absolute frequency,* % *relative frequency

Table 3 presents the distribution of study variable levels by gender. Overall, patterns were broadly similar between male and female participants, with no marked gender differences in the distributions of life satisfaction, self-esteem, or psychological well-being. These gender-based comparisons are exploratory and descriptive in nature, as no inferential statistical tests for gender differences were conducted.

**Table 3:** Descriptive analysis of the study variables by gender

Variable	Level	Gender	*n*	%
Life Satisfaction	Very dissatisfied	Female	34	6.7
		Male	27	5.3
	Dissatisfied	Female	54	10.7
		Male	62	12.3
	Neutral	Female	33	6.5
		Male	20	4.0
	Satisfied	Female	91	18.0
		Male	92	18.2
	Very satisfied	Female	57	11.3
		Male	36	7.1
Self-Esteem	Low	Female	0	0.0
		Male	1	0.2
	Medium-low	Female	59	11.7
		Male	47	9.3
	Medium-high	Female	145	28.7
		Male	132	26.1
	High	Female	65	12.8
		Male	57	11.3
Psychological Well-Being Levels	Low	Female	51	10.1
		Male	55	10.9
	Medium	Female	141	27.9
		Male	112	22.1
	High	Female	77	15.2
		Male	70	13.8

*n  *absolute frequency,* % *relative frequency

Table 4 presents the model fit indices for the hierarchical regression analysis. Full regression coefficients are presented in Table 5.

**Table 4:** Hierarchical Regression Analysis of life satisfaction on sociodemographic variables, psychological well-being dimensions, and self-esteem

Model	Predictors	*R*	*R*²	Adjusted *R*²	ΔR²	F	df1	df2	*p*
1	Sociodemographic variables	0.116	0.013	0.004	-	1.355	5	500	0.24
2	Psychological well-being dimensions	0.662	0.439	0.428	0.426	43.05	9	496	< 0.001
3	Self-esteem	0.772	0.596	0.588	0.157	73.14	10	495	< 0.001

*R² *coefficient of determination*; ΔR² *change in R²,* F  *F-statistic,* df  *degrees of freedom

Table 5 presents the hierarchical regression analysis, which revealed that sociodemographic variables alone did not significantly predict life satisfaction (Model 1, *R*^*2*^ = 0.013, *F* (5, 500) = *1.355*,*p* = 0.240). The addition of psychological well-being dimensions in Model 2 produced a substantial and statistically significant increment in explained variance (*ΔR² = 0.426*,*p* < 0.001), with social bonds emerging as the strongest predictor (*B* = 0.763, SE = 0.099, *β* = 0.333, *p* < 0.001), followed by projects (*B* = 0.463, SE = 0.091, *β* = 0.234, *p*< 0.001), acceptance/control (*B* = 0.411, SE = 0.119, *β* = 0.150, *p* < 0.001), and autonomy (*B* = 0.239, SE = 0.119, *β* = 0.086, *p* = 0.044). The inclusion of self-esteem in Model 3 yielded a further significant increment (*ΔR*²=0.157, *p*<0.001), with the final model accounting for 59.6% of the variance in life satisfaction (*R²=0.596*, adjusted *R²=0.588*). Self-esteem showed the strongest association with life satisfaction in the final model (*B* = 0.363, SE = 0.026, *β* = 0.572, *p* < 0.001). The standardized coefficient for self-esteem (*β* = 0.572) indicates that it accounts for the largest unique proportion of variance in life satisfaction among all predictors, suggesting that students’ global self-evaluation is the most influential psychological correlate of their subjective life appraisals. Its inclusion was associated with the attenuation of the effects of autonomy, projects, and acceptance/control to non-significance, while social bonds retained significance (*B = 0.424*, SE = 0.087, *β = 0.185*, *p* < 0.001), suggesting a potential mediating role of self-esteem.

**Table 5:** Hierarchical regression coefficients and model fit statistics predicting life satisfaction

							95% CI	
	**Predictor**	***B***	**SE**	***β***	**t**	*p*	lower	upper
Model 1	Intercept	17.454	1.698		10.278	< 0.001	14.126	20.782
	Age	-0.064	0.074	-0.041	-0.862	0.389	−0.209	0.08
	Gender	-0.45	0.395	-0.051	-1.139	0.255	−1.224	0.324
	University (private)	-0.386	0.469	-0.041	-0.823	0.411	−1.305	0.533
	University (other)	0.221	0.506	0.023	0.438	0.662	−0.771	1.213
	Place of residence	0.716	0.487	0.065	1.471	0.142	−0.239	1.671
Model 2	Intercept	4.567	1.465		3.117	0.002	1.696	7.438
	Age	-0.049	0.056	-0.032	-0.879	0.38	−0.159	0.06
	Gender	0.02	0.302	0.002	0.066	0.947	−0.572	0.612
	University (private)	-0.387	0.357	-0.041	-1.085	0.279	−1.087	0.313
	University (other)	-0.309	0.389	-0.032	-0.795	0.427	−1.071	0.453
	Place of residence	0.777	0.369	0.071	2.103	0.036	0.054	1.5
	Autonomy	0.239	0.119	0.086	2.019	0.044	0.006,	0.472
	Social bonds	0.763	0.099	0.333	7.735	< 0.001	0.569	0.957
	Projects	0.465	0.091	0.234	5.09	< 0.001	0.287	0.643
	Acceptance/Control	0.411	0.119	0.15	3.44	< 0.001	0.178	0.644
Model 3	Age	-0.012	0.048	-0.008	-0.255	0.799	−0.106	0.082
	Gender	-0.229	0.257	-0.026	-0.891	0.374	−0.733	0.275
	University (private)	-0.271	0.303	-0.029	-0.896	0.371	−0.865	0.323
	University (other)	-0.2	0.33	-0.021	-0.607	0.544	−0.847	0.447
	Place of residence	0.376	0.315	0.034	1.195	0.233	−0.241	0.993
	Acceptance/Control	0.177	0.103	-0.013	1.72	0.086	−0.025	0.379
	Autonomy	-0.037	0.103	0.185	-0.36	0.719	−0.239	0.165
	Projects	0.119	0.081	0.06	1.462	0.144	−0.040	0.278
	Social bonds	0.424	0.087	0.065	4.858	< 0.001	0.254,	0.594
	Self-esteem	0.363	0.026	0.572	13.913	< 0.001	0.312	0.414

*β  *standardized coefficient,* SE *standard error*.* Intercept values are reported for completeness, but are not interpreted substantively

**Fig. 1:**
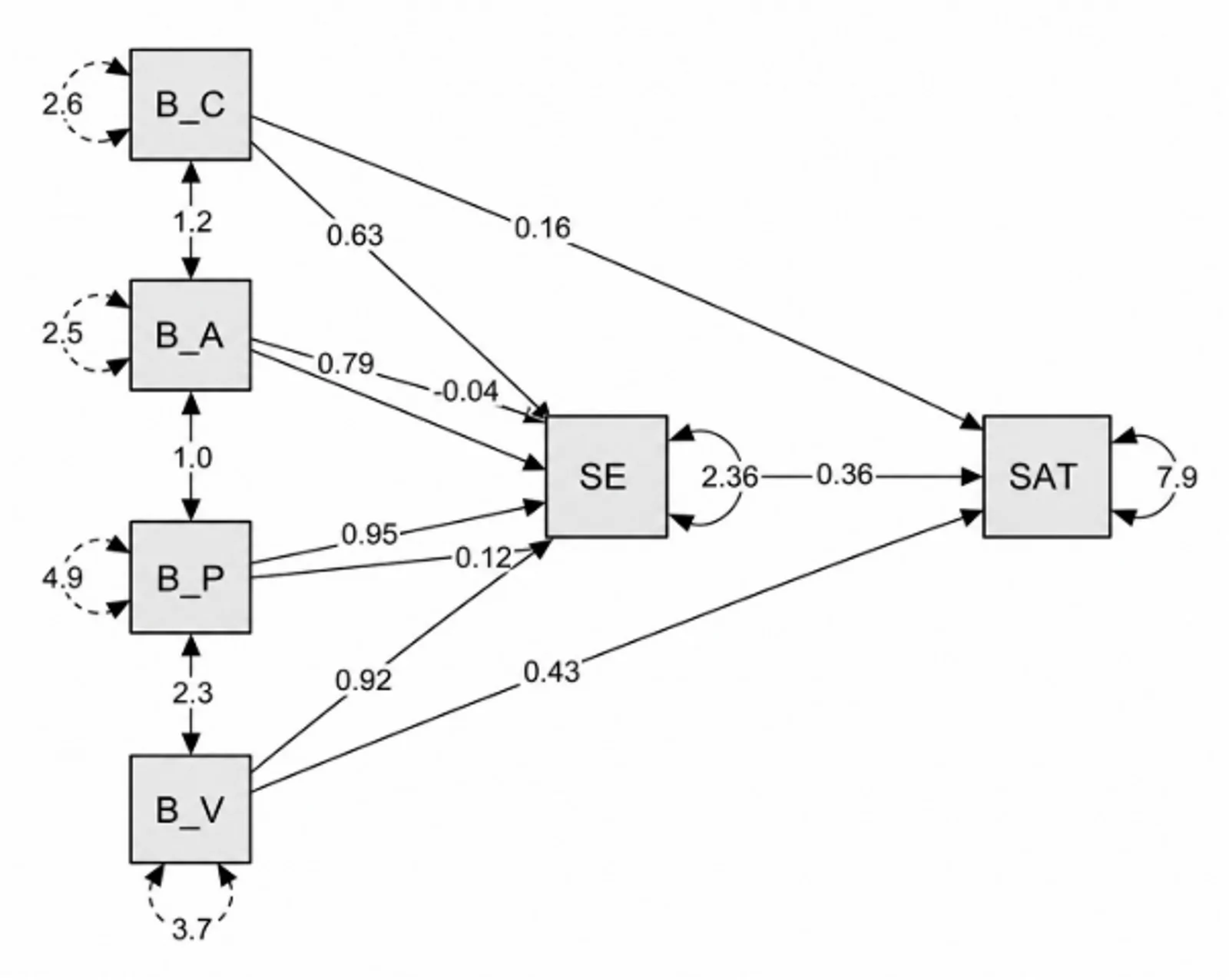
Path diagram on the mediation model examining self-esteem as a mediator between psychological well-being dimensions and life satisfaction among university students in southern Peru. Note: SAT = life satisfaction; SE = Self-esteem; B_C= Acceptance/Control; B_A=Autonomy; B_P = Life projects; B_V=Social Bonds. Numbers on arrows represent unstandardized coefficients (B). Solid arrows indicate statistically significant paths. Dashed arrows indicate non-significant paths

Table 6 presents the mediation analysis, which revealed that all four psychological well-being dimensions were significantly associated with self-esteem (path a), with projects showing the strongest association (*B* = 0.954, *p* < 0.001), followed by social bonds (*B* = 0.919, *p* < 0.001), autonomy (*B* = 0.789, *p* < 0.001), and control (*B* = 0.629, *p* < 0.001). Self-esteem, in turn, showed a strong and significant association with life satisfaction after controlling for all well-being dimensions (path b: *B* = 0.365, *p* < 0.001). Regarding direct effects (path c’), only social bonds retained a significant association with life satisfaction (*B* = 0.433, *p* < 0.001), while control (*p* = 0.132), autonomy (*p* = 0.686), and projects (*p* = 0.174) became nonsignificant, with confidence intervals crossing zero. These results indicate full mediation for control, autonomy, and projects, and partial mediation for social bonds, supporting self-esteem as a central psychological mechanism linking well-being dimensions to life satisfaction (Fig. 1). Furthermore, all four indirect effects (a x b) were statistically significant, with bootstrap confidence intervals excluding zero. The path model showed an adequate fit to the data, CFI = 0.996, TLI = 0.988, RMSEA = 0.046, 90% CI [0.000, 0.098], SRMR = 0.014.

**Table 6:** Self-esteem as a mediating mechanism between well-being dimensions and life satisfaction among university students in southern Peru

			Estimate	Std. error	z-value	*p*	*95% Confidence Interval*	
							Lower	Upper
Self-esteem	→	Life satisfaction	0.365	0.027	13.29	< 0.001	0.312	0.419
Control	→	Life satisfaction	0.159	0.106	1.506	0.132	-0.066	0.358
Autonomy	→	Life satisfaction	-0.042	0.104	-0.404	0.686	-0.226	0.185
Projects	→	Life satisfaction	0.123	0.090	1.358	0.174	-0.041	0.311
Social bonds	→	Life satisfaction	0.433	0.096	4.517	< 0.001	0.226	0.600
Control	→	Self-esteem	0.629	0.163	3.870	< 0.001	0.334	0.978
Autonomy	→	Self-esteem	0.789	0.167	4.739	< 0.001	0.473	1.121
Projects	→	Self-esteem	0.954	0.142	6.722	< 0.001	0.681	1.237
Social bonds	→	Self-esteem	0.919	0.148	6.213	< 0.001	0.616	1.197
**Indirect effects ( axb)**								
Control	→	Life satisfaction	0.23	0.063	3.663	< 0.001	0.111	0.359
Bonding	→	Life satisfaction	0.335	0.056	5.959	< 0.001	0.162	0.427
Projects	→	Life satisfaction	0.348	0.056	6.164	< 0.001	0.244	0.463
Autonomy	→	Life satisfaction	0.288	0.067	4.294	< 0.001	0.231	0.450
Total effects (c’ + a×b)								
Control			0.389	0.111	3.504	< 0.001	0.17	0.605
Bonding			0.768	0.102	7.53	< 0.001	0.564	0.973
Projects			0.471	0.084	5.592	< 0.001	0.306	0.636
Autonomy			0.246	0.109	2.259	0.024	0.034	0.461

*B*  Unstandardized coefficient, *SE*  Standard error, *z*  z-value, *CI*  confidence interval. Path a = effect of psychological well-being dimensions on self-esteem- path b = effect of self-esteem on life satisfaction, controlling for well-being dimensions. Path c’ = direct effect of well-being dimensions on life satisfaction, controlling for self-esteem

## Discussion

The main objective of the study was to determine whether self-esteem and psychological well-being dimensions predict life satisfaction among university students in southern Peru. The hierarchical regression analysis revealed that sociodemographic variables alone failed to predict life satisfaction. In contrast, the sequential inclusion of psychological well-being dimensions and self-esteem produced substantial increments in explained variance, with the final model accounting for 59.6% of the variance in life satisfaction. This pattern indicates that subjective and psychological factors far outweigh demographic characteristics in shaping how university students evaluate their lives, consistent with Diener’s [13] proposition that life satisfaction is primarily governed by internal appraisal processes rather than objective life circumstances.

These findings are further interpreted in light of Ryff’s multidimensional model of psychological well-being [15] and Diener’s subjective well-being framework [13]. While Ryff’s model emphasizes psychological well-being as a multidimensional construct encompassing autonomy, purpose, self-acceptance, environmental mastery, personal growth, and positive relations, Diener’s framework focuses on the cognitive evaluation of life as a whole. The present results suggest that self-esteem operates as a linking mechanism between these two theoretical perspectives, translating psychological well-being capacities into subjective life evaluations, a mediating role not explicitly articulated in either model’s original formulation.

Self-esteem emerged as the strongest predictor of life satisfaction in the final model (*β* = 0.572, *p* < 0.001), surpassing all psychological well-being dimensions. This finding may be explained by the proximal nature of self-evaluative processes, whereas psychological well-being captures broader functional dimensions; self-esteem operates as a direct appraisal of one’s own worth that more immediately shapes subjective life evaluations [40, 52].

The path analysis confirmed that all four psychological well-being dimensions exerted their effects on life satisfaction primarily through self-esteem, reinforcing the notion that self-evaluative processes constitute a central psychological mechanism through which Ryff’s dimensions are translated into subjective life appraisal [53, 54]. However, the magnitude of these indirect effects varied across dimensions, underscoring the importance of examining psychological well-being as a multidimensional rather than a unidimensional construct. This variation likely reflects how closely each dimension relates to global self-worth. Projects and social bonds showed the strongest paths to self-esteem, autonomy showed an intermediate path, and control showed the weakest, suggesting that purpose and social connectedness contribute most directly to self-evaluations, while personal control contributes comparatively less.

Among the dimensions examined, social bonds showed the strongest direct association with life satisfaction (*β* = 0.189, *p* < 0.001), whereas autonomy and control did not reach statistical significance when self-esteem was included in the model. The psychological well-being dimensions also yielded statistically significant independent associations with life satisfaction, although the magnitude of these effects was smaller than that of self-esteem, consistent with prior evidence demonstrating that functioning and subjective well-being, while positively correlated, represent empirically distinct constructs [11]. This differential pattern is particularly meaningful in southern Peru’s collectivist sociocultural context, where interpersonal bonds carry intrinsic cultural value, which may account for why social bonds retained a statistically significant direct association with life satisfaction independent of self-esteem. These findings are consistent with self-determination theory [11], which identifies relatedness as a fundamental psychological need whose satisfaction contributes directly to well-being. Beyond social bonds, southern Peru’s structural conditions: geographic dispersion, and limited access to health services may also constrain opportunities for autonomy and environmental mastery relative to more resource-rich settings. This is illustrated by evidence from indigenous Quechua- and Aymara-speaking university students in the Arequipa region, who report social isolation linked to language barriers and pressure to assimilate into a Spanish-speaking academic environment [55], underscoring how structural and cultural conditions in southern Peru may shape dimensions of well-being tied to individual agency. These findings are consistent with previous research documenting positive associations between self-esteem and life satisfaction across diverse university populations. Studies conducted in Peru, Mexico, Bolivia, and Spain have reported that individuals with higher self-esteem tend to report greater life satisfaction [24, 37, 44], with self-esteem showing significant associations with stronger self-concept, greater self-efficacy, and more effective emotional regulation [38–40, 50]. A study involving 452 university students in Chile further demonstrated that psychological distress was inversely associated with both self-esteem and life satisfaction [41], reinforcing the notion that self-esteem is more closely related to subjective appraisals and lived experience than to external circumstance, and that its contribution to life satisfaction operates independently of sociodemographic factors such as age, gender, employment status, educational level, or marital status [42, 45]. Evidence from Ecuador similarly showed that higher psychological well-being was positively associated with greater life satisfaction [43]. Educational activities have further been found to strengthen self-concept and promote holistic student well-being [46].

The mediation analysis indicated that self-esteem mediated the associations between psychological well-being dimensions and life satisfaction, with full mediation observed for control, autonomy, and projects, and partial mediation for social bonds. Specifically, the indirect effects of control, autonomy, projects, and social bonds were all statistically significant, with confidence intervals excluding zero. These results suggest that psychological well-being dimensions contribute to life satisfaction primarily by strengthening self-evaluative processes rather than through direct pathways. A study found that both personal and relational self-esteem mediated the association between hope and life satisfaction among university students [56]. Further evidence indicates that self-esteem mediates the association between empathy and life satisfaction [50], between health-related quality of life and life satisfaction in low-income populations [57], and between well-being literacy and subjective well-being [58]. Collectively, these findings reinforce the notion that self-esteem constitutes a core psychological mechanism through which diverse individual resources are internalized and translated into positive life evaluations.

Gender differences in self-esteem and life satisfaction have yielded inconsistent findings in the literature: while some studies report higher self-esteem in men [47, 49], others report no statistically significant differences [24]. Descriptive analyses in the present study indicated that men showed a higher concentration at the satisfied level of life satisfaction (18.2%), while women predominated at the medium-high level of self-esteem (28.7%) and the medium level of psychological well-being (27.9%), which is similar to other reports showing a strong positive relationship between self-esteem and life satisfaction among female university students [48]. However, regression analyses did not yield a statistically significant gender coefficient (*β* = -0.026, *p* = 0.374), which may reflect the demographic homogeneity of the sample, predominantly single young adults enrolled in engineering programs, or shared structural barriers across gender groups in this sociocultural context.

This study has certain limitations that should be considered when interpreting the findings. The use of non-probabilistic convenience sampling limits external validity, as the sample may not represent the broader university student population; this may have introduced selection bias that could affect the generalizability of the findings to other sociocultural contexts. Furthermore, the predominance of engineering students in the sample may somewhat limit generalizability even within southern Peru, as engineering programs can differ from health, social, and education sciences in academic demands, gender composition, and stress profiles. The cross-sectional design limits internal validity by precluding causal inference; the directionality of the observed associations cannot be established, and therefore the findings should be interpreted as associations rather than causal effects. Reliance on self-report measures may introduce common method variance, inflating observed associations among constructs and affecting the reliability of the findings. In addition, this study did not assess socioeconomic status, which is a limitation. Students from lower socioeconomic backgrounds may have participated less due to limited access to the internet or devices, and may have shown different levels of self-esteem and well-being; this could have altered the strength of the associations reported here.

Future research should extend these findings using longitudinal designs to clarify the temporal relationship among psychological well-being dimensions, self-esteem, and life satisfaction. Experimental studies could further examine whether interventions aimed at enhancing self-esteem improve life satisfaction. Additionally, future studies should incorporate moderating variables such as social support, socioeconomic status, and academic stress to better understand the contextual conditions influencing the relationship. Extending the model to include additional mediators, such as emotional regulation, resilience, and sense of belonging, would contribute to a more comprehensive understanding of the psychological mechanisms by which well-being dimensions shape life satisfaction.

Grounded in Ryff’s framework and Diener’s subjective well-being model, the present study provides empirical evidence that self-esteem is a key mediating mechanism linking dimensions of psychological well-being to life satisfaction among university students in southern Peru. These findings underscore the importance of designing interventions that simultaneously address psychological well-being dimensions as key personal resources and self-evaluative processes to promote life satisfaction in university populations facing structural and psychological challenges.

## Conclusions

The results confirm that self-esteem and psychological well-being are significant cross-sectional predictors of life satisfaction among university students in southern Peru, with self-esteem emerging as the strongest predictor and serving as a key mediating mechanism linking the dimensions of psychological well-being to life satisfaction. These findings contribute to the international literature on positive psychology by demonstrating that self-esteem mediates the relationship between psychological well-being and subjective well-being in a Latin American university context that remains underrepresented in the global psychological literature, thereby addressing a meaningful gap in the international evidence base.

## Data Availability

No datasets were generated or analysed during the current study.

## Abbreviations

BIEPS-A: Psychological Well-Being Scale for Adults
DPT: Predictive and Cross-Sectional Design
